# Role of Autophagy in Zinc Oxide Nanoparticles-Induced Apoptosis of Mouse LEYDIG Cells

**DOI:** 10.3390/ijms20164042

**Published:** 2019-08-19

**Authors:** Jingcao Shen, Dan Yang, Xingfan Zhou, Yuqian Wang, Shichuan Tang, Hong Yin, Jinglei Wang, Rui Chen, Jiaxiang Chen

**Affiliations:** 1 Department of Physiology, Medical College of Nanchang University, Nanchang 330006, China; 2 Key Laboratory of Occupational Health and Safety, Beijing Municipal Institute of Labor Protection, Beijing 100054, China; 3 School of Aerospace, Mechanical and Manufacturing Engineering, RMIT University, Bundoora, VIC 3083, Australia; 4 Jiangxi Provincial Key Laboratory of Reproductive Physiology and Pathology, Nanchang 330006, China

**Keywords:** ZnO NPs, Leydig cells, apoptosis, autophagy, oxidative stress

## Abstract

Zinc oxide nanoparticles (ZnO NPs) have shown adverse health impact on the human male reproductive system, with evidence of inducing apoptosis. However, whether or not ZnO NPs could promote autophagy, and the possible role of autophagy in the progress of apoptosis, remain unclear. In the current study, in vitro and in vivo toxicological responses of ZnO NPs were explored by using a mouse model and mouse Leydig cell line. It was found that intragastrical exposure of ZnO NPs to mice for 28 days at the concentrations of 100, 200, and 400 mg/kg/day disrupted the seminiferous epithelium of the testis and decreased the sperm density in the epididymis. Furthermore, serum testosterone levels were markedly reduced. The induction of apoptosis and autophagy in the testis tissues was disclosed by up-regulating the protein levels of cleaved Caspase-8, cleaved Caspase-3, Bax, LC3-II, Atg 5, and Beclin 1, accompanied by down-regulation of Bcl 2. In vitro tests showed that ZnO NPs could induce apoptosis and autophagy with the generation of oxidative stress. Specific inhibition of autophagy pathway significantly decreased the cell viability and up-regulated the apoptosis level in mouse Leydig TM3 cells. In summary, ZnO NPs can induce apoptosis and autophagy via oxidative stress, and autophagy might play a protective role in ZnO NPs-induced apoptosis of mouse Leydig cells.

## 1. Introduction

Nanotechnology manipulates matters at the atomic, molecular, and supramolecular scales and has grown rapidly worldwide in the past decades. With the development of nanotechnology, environmental exposure to nanoparticles (NPs) is increasing dramatically [1,2]. Metal oxide nanoparticles are the most abundantly produced types of engineered nanomaterials in industry [3]. Among them, zinc oxide nanoparticles (ZnO NPs) are used in various applications, such as cosmetics, rubber manufacture, pigments, food additives, biosensors, chemical fibers, bioimaging, and antibacterial agents, due to their low production cost and unique physicochemical properties [4]. ZnO NPs may enter human bodies by various routes, including inhalation, dermal penetration, injection, and ingestion [5]. These NPs can then accumulate in various organs, such as the liver, spleen, lungs, kidney, and heart via circulation, and may produce adverse consequences, such as edema and degeneration of hepatocytes, inflammation of the pancreas, or damage to the stomach and spleen [6,7]. Consequently, toxicity research and health risk assessments of ZnO NPs have attracted tremendous attention recently [8].

Tissue damage due to NPs exposure arises from direct cell–NPs interaction and is associated with local concentrations of exogenous substances in the tissues, i.e., the nanoparticle itself or solubilized ions [9,10]. Previous researches show that zinc ion dissolved from the surface of ZnO NPs is a primary reason for its cytotoxicity [11,12]. Reducing the ion release from the surface, such as by pre-coating with a protein corona, could greatly decrease their cytotoxicity [13,14]. Furthermore, our previous research illustrates that ZnO NPs and their soluble ions can induce significant cellular endoplasmic reticulum (ER) stress responses before triggering ER-related apoptosis [12]. Generation of reactive oxygen species (ROS) is generally involved in cellular damage from the exposure to ZnO NPs [12,13,15]. ROS are chemically-reactive molecules containing oxygen, which are generated as by-products of biological oxidation during mitochondrial respiration under physiological conditions. ROS include both free radicals, such as nitric oxide (NO), superoxide (O_2_^•−^), and hydroxyl radical (^•^OH), and peroxides [16]. Reduced glutathione (GSH) and antioxidant enzymes such as glutathione peroxidase (GSH-PX) and superoxide dismutase (SOD) are normally used to scavenge ROS. Oxidative stress occurs when there is an imbalance between ROS production and the cellular antioxidant defence system [17,18].

Spermatogenesis consists of highly organized and sequential steps of undifferentiated spermatogonial stem cell proliferation and differentiation, which generates functional sperms in the testis [19,20,21]. ZnO NPs can cause vacuolization of germinal epithelium and sloughing of germ, and even decrease the sperm number and motility in the epididymis [22]. In addition to Sertoli cells, Leydig cells play an important role in maintaining spermatogenesis and are prone to being affected by various chemicals [23,24]. ZnO NPs have been reported to exert cytotoxic effects on mouse Leydig cells [25,26]. Similar toxic effects were revealed in the testis of six-month-old common carp Cyprinus carpio after exposure to 10, 50, and 100 μg/L ZnO NPs for 21 days [27]. Furthermore, increasing evidence suggests that the toxicity of ZnO NPs may result from ROS production [9,28,29].

Autophagy is an evolutionarily-conserved, highly-regulated lysosomal degradative pathway involving the delivery of cytoplasmic cargo to the lysosome, which occurs at low basal levels to perform protein and organelle turnover in normal situations [30,31]. Autophagy can be induced during starvation or growth factor withdrawal in order to generate more intracellular nutrients and energy [32]. Autophagy can also be induced under stressful conditions such as neurodegenerative diseases, pathogen infections, chemotherapy, and chemical exposure [33,34,35,36,37]. Increasing evidence has shown that ZnO NPs can induce autophagy in immune cells, normal skin cells, gastrointestinal tract cells, and kidney tissue [7,38,39,40]. Until now, there has been no evidence that ZnO NPs exposure could induce autophagy in testis tissue.

The aims of the present study were to investigate whether oxidative stress was involved in ZnO NPs-induced apoptosis and autophagy of mouse Leydig cells, and to determine the role of autophagy in ZnO NPs-induced apoptosis. These results will provide fundamental understanding of ZnO NPs-induced spermatogenesis failure.

## 2. Results

### 2.1. Characteristics and Morphology of ZnO NPs

Transmission electron microscopy (TEM) test shows the primary size of ZnO NPs is about 30 nm with a propensity to agglomerate (Figure S2). These characteristics are comparable to previous publications using the same nanoparticles [41,42]. The hydrodynamic sizes and zeta potentials of ZnO NPs suspended in water are 66.36 ± 0.39 nm (PDI = 0.167, *n* = 3) and 38.25 ± 1.06 mV (*n* = 3), respectively.

### 2.2. ZnO NPs Cause Testis Damage to Male Mice

**Figure 1:**
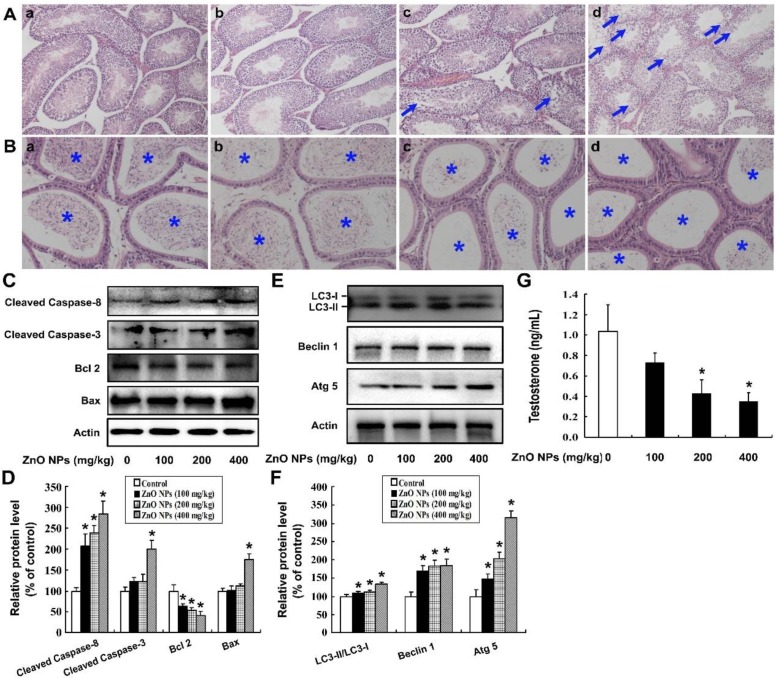
Intragastrical exposure of zinc oxide nanoparticles (ZnO NPs) cause toxic damage to the mouse male reproductive system. (**A**) Testes were obtained from male mice treated with 0 (**a**), 100 (**b**), 200 (**c**), or 400 (**d**) mg ZnO NPs/kg/day for 28 days. The testes were stained with hematoxylin and eosin (HE) and then were visualized under an IX51 Olympus microscope. The disruption of the seminiferous epithelium in the testis is indicated by arrows. Magnification: 100×. (**B**) Epididymides were obtained from male mice treated with 0 (**a**), 100 (**b**), 200 (**c**), or 400 (**d**) mg ZnO NPs/kg/day for 28 days, and stained with HE. The sperm in the epididymis are indicated by an asterisk. Magnification: 200×. (**C**) The protein levels of cleaved Caspase-3, cleaved Caspase-8, Bax, and Bcl 2 and (**E**) the levels of LC3, Beclin 1, and Atg 5 were detected by Western blot; Actin was used as an internal control. (**D,F**) The relative protein levels were quantified by densitometry. (**G**) The serum testosterone concentration. The experiment was done in triplicate and repeated three times (*n* = 9). Data were analyzed by one-way ANOVA. * *p* < 0.05.

As shown in Figure 1A, the testes of vehicle-treated mice showed normal seminiferous tubules lined with both spermatogenic cells and Sertoli cells. No detached germ cells were found in the tubular lumen. In the 100 mg/kg/day ZnO NPs exposure group, no significant morphologic changes were observed at the seminiferous epithelium. However, the seminiferous tubule demonstrated mildly disorganized histo-architecture in the 200 mg/kg/day group. In the 400 mg/kg/day group, seminiferous tubules exhibited disintegration of the germinal epithelium, germ cell depletion, and a reduction in round sperm. There was a significant decrease in sperm density of the epididymis after exposure to 100, 200, or 400 mg ZnO NPs/kg/day compared to the vehicle control group (Figure 1B), indicating that ZnO NPs exposure significantly inhibited spermatogenesis.

To further investigate the potential mechanism of ZnO NPs-induced spermatogenesis failure, the apoptosis level in the mouse testis tissues was assessed. As can be seen from Figure 1C,D, ZnO NPs significantly increased the levels of apoptosis-related proteins, including cleaved Caspase-8, cleaved Caspase-3 and Bax, along with a decreased protein level of Bcl 2 in the testis tissue, which indicates that ZnO NPs induced apoptosis of the testis tissue. Additionally, ZnO NPs markedly increased the ratio of LC3-II/LC3-I, as well as the levels of autophagy proteins Atg 5 and Beclin 1, indicating that ZnO NPs induced autophagy of the testis tissue (Figure 1E,F). Furthermore, ZnO NPs decreased the serum testosterone concentration in a dose-dependent manner (*p* < 0.05), which implies that ZnO NPs disrupted the physiological function of the male reproductive system by targeting the Leydig cells (Figure 1G).

### 2.3. ZnO NPs Induce Apoptosis of Mouse Leydig TM3 Cells

**Figure 2:**
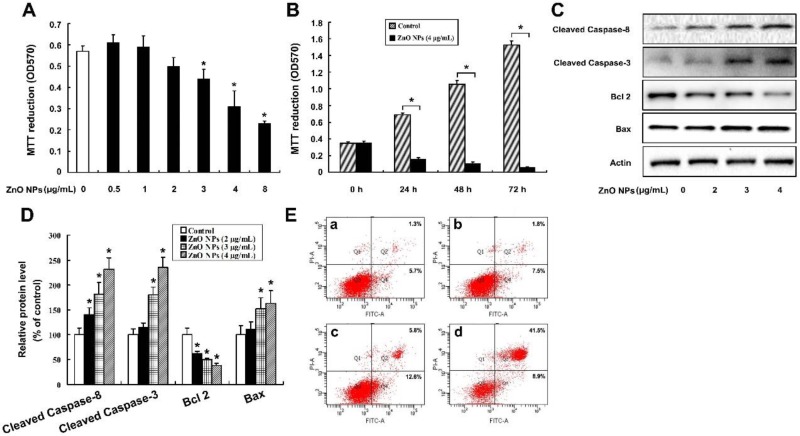
ZnO NPs induce apoptosis in mouse Leydig TM3 cells. 3-(4,5-dimethylthiazol-2-yl)-2,5-diphenyltetrazolium bromide (MTT) assay results of mouse Leydig TM3 cells treated with 0–8 μg/mL ZnO NPs for 24 h (**A**) or treated with 4 μg/mL ZnO NPs for 24~72 h (**B**). (**C**) The cells were treated with 0–4 μg/mL ZnO NPs for 24 h; then, the protein levels of cleaved Caspase-3, cleaved Caspase-8, Bcl 2, and Bax were investigated by Western blot; Actin was used as an internal control. (**D**) The relative protein levels were quantified by densitometry. (**E**) The cells were treated with 0 (**a**), 2 (**b**), 3 (**c**), 4 (**d**) μg/mL ZnO NPs for 24 h, then the AnnexinV-FITC positive staining cells were counted by flow cytometry. The experiment was done in triplicate and repeated three times. Data were analyzed by one-way ANOVA. * *p* < 0.05.

The content of testosterone dramatically decreased in the ZnO NPs-treated groups, which implies that ZnO NPs might cause damage to Leydig cells. To further verify the hypothesis, mouse Leydig TM3 cell line was utilized as an in vitro model. As shown in Figure 2A, ZnO NPs at concentrations of 3, 4, and 8 μg/mL significantly inhibited cell viability. Further tests showed that the cell viability was further suppressed at time points of 24, 48, and 72 h post-exposure to 4 μg/mL ZnO NPs (Figure 2B). To determine whether the anti-proliferative effect of ZnO NPs resulted from apoptosis, the apoptosis-related proteins were investigated, including cleaved Caspase-8, cleaved Caspase-3, Bcl 2 and Bax, after the cells were incubated with 0, 2, 3, and 4 μg/mL ZnO NPs for 24 h. It was shown that ZnO NPs dramatically increased the protein levels of cleaved Caspase-8, cleaved Caspase-3, and Bax, as well as decreased Bcl 2 protein level (Figure 2C,D). Furthermore, ZnO NPs increased the numbers of AnnexinV-FITC positive staining cells (Figure 2E). These results indicate that ZnO NPs induced apoptosis of mouse Leydig TM3 cells.

### 2.4. ZnO NPs Induce Apoptosis through Activation of Oxidative Stress

**Figure 3:**
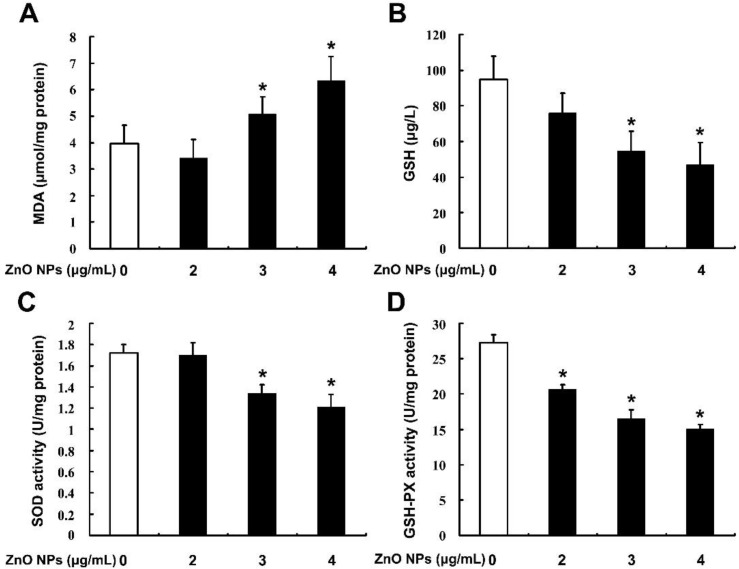
ZnO NPs induce oxidative stress in mouse Leydig TM3 cells. Mouse Leydig TM3 cells were treated with 0–4 μg/mL ZnO NPs for 24 h; then the contents of MDA (**A**) and GSH (**B**) and the enzyme activities of SOD (**C**) and GSH-PX (**D**) were determined. The experiment was done in triplicate and repeated three times. Data were analyzed by one-way ANOVA. * *p* < 0.05.

**Figure 4:**
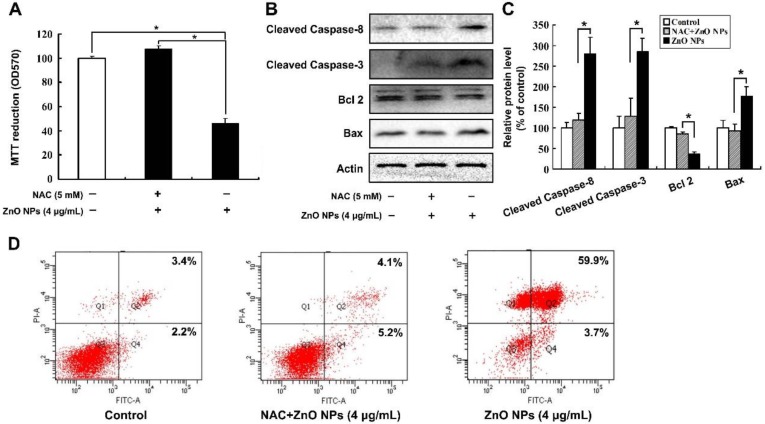
Oxidative stress is involved in ZnO NPs-induced apoptosis of mouse Leydig TM3 cells. Mouse Leydig TM3 cells were treated with 4 μg/mL ZnO NPs for 24 h in the absence or presence of 5 mM NAC, then cell viability (**A**), the protein levels of cleaved Caspase-8, cleaved Caspase-3, Bcl 2, and Bax (**B**) and the AnnexinV-FITC positive staining cells (**D**) were detected by MTT assay, Western blot, and flow cytometry, respectively. (**C**) The relative protein levels of cleaved Caspase-8, cleaved Caspase-3, Bcl 2, and Bax were quantified by densitometry. The experiment was done in triplicate and repeated three times. Data were analyzed by one-way ANOVA. * *p* < 0.05.

In order to investigate whether oxidative stress was involved in ZnO NPs-induced apoptosis of mouse Leydig TM3 cells, the contents of malondialdehyde (MDA) and GSH and the enzyme activities of SOD and GSH-PX were determined after the cells were treated with ZnO NPs for 24 h. As shown in Figure 3, ZnO NPs significantly increased MDA level in the cells in a dose-dependent manner, whereas the content of GSH and the activities of the antioxidant enzymes SOD and GSH-PX were decreased in the ZnO NPs-treated cells, which implies that ZnO NPs induced oxidative stress in mouse Leydig TM3 cells. The same markers were detected after the cells were treated with H_2_O_2_ and the cell viability was significantly inhibited with the induction of apoptosis, suggesting that oxidative stress could induce apoptosis of mouse Leydig TM3 cells (Figure S3). The mouse Leydig TM3 cells were treated with 4 μg/mL ZnO NPs for 24 h in the presence or absence of 5 mM NAC, an inhibitor of ROS, to further confirm the role of oxidative stress in ZnO NPs-induced apoptosis. As shown in Figure 4, inhibition of viability and induction of apoptosis by ZnO NPs was significantly rescued by NAC. These results illustrate that oxidative stress was involved in ZnO NPs-induced apoptosis of mouse Leydig TM3 cells.

### 2.5. Oxidative Stress is Involved in ZnO NPs-Induced Autophagy

**Figure 5:**
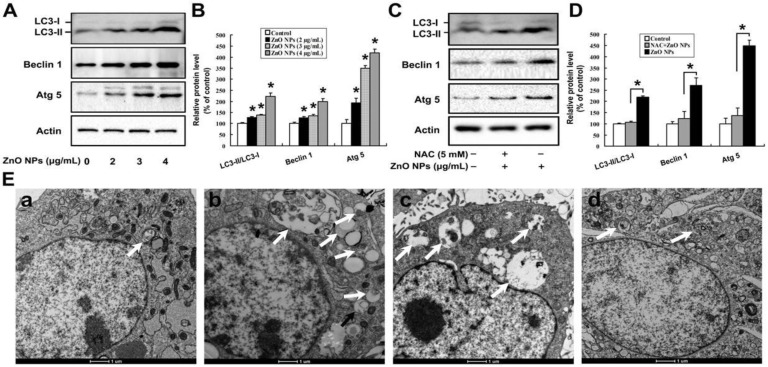
Oxidative stress is involved in ZnO NPs-induced autophagy of mouse Leydig TM3 cells. Mouse Leydig TM3 cells were treated with 0–4 μg/mL ZnO NPs for 24 h (**A**) or treated with 4 μg/mL ZnO NPs for 24 h in absence or presence of 5 mM NAC (**C**); then, the protein levels of LC 3, Atg 5, and Beclin 1 were quantified by Western blot. (**B,D**) The relative protein levels of LC 3, Atg 5, and Beclin 1 were quantified by densitometry. (**E**) The cells were treated with ddH_2_O, bars: 1 μm, (**a**), 4 μg/mL ZnO NPs (**b**), or 5 mM N-acetyl-L-cysteine (NAC) plus 4 μg/mL ZnO NPs for 24 h (**d**). Then, autophagic vacuoles in the cells were visualized by transmission electron microscopy (TEM), with starvation-treated cells as a positive control (**c**). The autophagic vacuoles are indicated by white arrows. The experiment was done in triplicate and repeated three times. Data were analyzed by one-way ANOVA. * *p* < 0.05.

As shown in Figure 5A,B, ZnO NPs increased the ratio of LC3-II to LC3-I, as well as the protein levels of Atg 5 and Beclin 1. Similarly, H_2_O_2_ markedly increased the ratio of LC3-II to LC3-I and the contents of Atg 5 and Beclin 1, indicating that oxidative stress could induce autophagy of mouse Leydig TM3 cells (Figure S4). Furthermore, inhibition of oxidative stress could rescue the induction of autophagy by ZnO NPs (Figure 5C,D). ZnO NPs-induced autophagy was further investigated by TEM. As shown in Figure 5E, there were relatively few autophagosomes in the cytoplasm of the control cells, while autophagic vacuoles containing extensively-degraded organelles (such as mitochondria and endoplasmic reticulum) significantly increased in both ZnO NPs-treated cells and starvation-treated cells. Interestingly, inhibition of oxidative stress decreased the number of autophagosomes. These results suggest that oxidative stress played an important role in ZnO NPs-induced autophagy. It is worthy to note that ZnO NPs-induced autophagy and apoptosis of mouse Leydig TM3 cells might be closely related to the soluble zinc ions, as similar bio-effects were observed after the cells were treated with 0–1 μg/mL ZnCl_2_ (Figure S5).

### 2.6. Inhibition of Autophagy Increases ZnO NP-Induced Apoptosis

**Figure 6:**
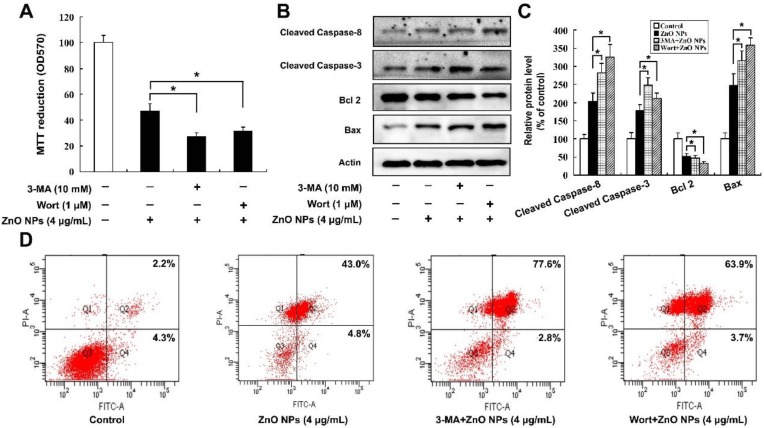
Inhibition of autophagy increases the ZnO NPs-induced apoptosis level in mouse Leydig TM3 cells. Mouse Leydig TM3 cells were treated with 4 μg/mL ZnO NPs for 24 h in the absence or presence of 10 mM 3-MA or 1 μM Wortmannin (Wort), then cell viability (**A**), the protein levels of cleaved Caspase-8, cleaved Caspase-3, Bcl 2, and Bax (**B**) and the AnnexinV-FITC positive staining cells (**D**) were tested by MTT assay, Western blot, and flow cytometry, respectively. (**C**) The relative protein levels of cleaved Caspase-8, cleaved Caspase-3, Bcl 2, and Bax were quantified by densitometry. The experiment was done in triplicate and repeated three times. Data were analyzed by one-way ANOVA. * *p* < 0.05.

As apoptosis and autophagy were both induced by ZnO NPs, the effects of autophagy on ZnO NPs-induced apoptosis were studied. Cell viability was measured after the cells were treated with 4 μg/mL ZnO NPs for 24 h in the absence or presence of an autophagy inhibitor, either 10 mM 3-Methyladenine (3-MA) or 1 μM Wortmannin (Wort). Compared with the ZnO NPs-treated cells, inhibition of autophagy further decreased viability of mouse Leydig TM3 cells (Figure 6A) and up-regulated the protein levels of cleaved Caspase-8, cleaved Caspase-3, and Bax, accompanied by the down-regulation of Bcl 2 protein (Figure 6B,C). The number of AnnexinV-FITC positive staining cells were also markedly increased when autophagy was inhibited (Figure 6D). These results indicate that autophagy might play a protective role in ZnO NPs-induced apoptosis of mouse Leydig TM3 cells.

## 3. Discussion

Exposure to ZnO NPs for humans is inevitable due to their wide applications in commercial and industrial products. Thus, the adverse effects from the exposure of ZnO NPs need clear definition. Nanoparticles can pass through the blood–brain barrier (BBB), blood–testis barrier (BTB), and blood–air barrier (BAB), with the ability to accumulate in the brain, the testis, or peripheral organs [43,44,45,46]. Recently, Qian et al. showed that ZnO NPs could cause adverse effects throughout the male reproductive system by impairing the BTB [47]. Oral dose toxicity has been reported in SD mice after repetitive exposure to positively-charged 100 nm ZnO NPs over 14 or 90 days, and the target organs were found to be the spleen, stomach, and pancreas, with a no-observed-adverse-effect dose level of about 125 mg/kg (b.w.) [48,49]. In our research, it was shown that 28-day gavage exposure of ZnO NPs (30 nm positively charged) at the concentrations of 100, 200, and 400 mg/kg/day caused disruption and atrophy of the seminiferous epithelium in the testis of mice. Furthermore, the sperm density in the epididymis significantly decreased in the ZnO NPs-treated groups, which was in good agreement with some previous work [22,27]. This toxic dosage range is also similar to the research of Hong et al., in which they tested the toxicity on embryo-fetal development in rats from 15 days of repeated oral doses of 20 nm negatively-charged ZnO NPs [50]. Therefore, ZnO NPs, with the high chance of daily contact and exposure, may pose a high risk of reproductive toxicity after long-term accumulation in the human body.

ZnO NPs have been shown to induce apoptosis in many cells such as human epidermal keratinocytes, human aortic endothelial cells, human liver and kidney podocytes [51,52,53,54]. Han et al. showed that ZnO NPs took cytotoxic effects on mouse testicular cells and induced apoptosis in Leydig cells [25]. In this research, we confirmed that ZnO NPs up-regulated the protein levels of Bax, cleaved Caspase-3, and cleaved Caspase-8 in the testis tissue, as well as decreased the protein level of Bcl 2, which indicates that ZnO NPs could induce apoptosis in the testis.

Autophagy protein LC3, a widely used marker of mammalian autophagy, has two forms, i.e., a cytosolic form (LC3-I) and an autophagic vesicle-associated form (LC3-II). During induction of autophagy, LC3-I covalently conjugates with phosphatidylethanolamine and develops LC3-II, which is recruited and bound to the autophagosome membrane [33,55]. The conversion of LC3-I to LC3-II is considered to be a crucial step in initiating autophagy [56], with the amount of LC3-II related to the extent of autophagosome formation [55]. In the present study, ZnO NPs exposure significantly increased the ratio of LC3-II to LC3-I in the testis tissue, along with similar up-regulation of autophagy proteins Atg 5 and Beclin 1. These results implied that ZnO NPs could induce autophagy in the testis tissue.

The primary function of Leydig cells is the synthesis and secretion of androgen, which plays an important role in spermatogenesis [57]. In our study, ZnO NPs could decrease serum testosterone level, indicating that Leydig cells might be the target for ZnO NPs-induced spermatogenesis failure. To further verify this hypothesis, the mouse Leydig TM3 cell line was utilized as an in vitro research model. In agreement with the in vivo findings, ZnO NPs exposure inhibited viability and induced apoptosis of mouse Leydig TM3 cells. Thus, it is reasonable to speculate that the inhibition of cell viability upon ZnO NPs exposure might result from the induction of apoptosis.

Oxidative stress has been identified as a critical pathophysiological mechanism of reproductive toxicity from environmental chemicals or organophosphorus compounds [58]. Asani et al. showed that ZnO NPs could induce oxidative stress in pancreatic β-cells [59]. Similar to the toxicity effects of H_2_O_2_, exposure to ZnO NPs significantly increased the MDA in the cells, along with a marked decrease in both the GSH levels and the enzyme activities of SOD and GSH-PX. Further evidence demonstrates that apoptosis could be distinctly reduced when oxidative stress was inhibited, which confirmed that oxidative stress was involved in ZnO NPs-induced apoptosis of mouse Leydig TM3 cells. Oxidative stress has been shown to induce autophagy and plays an important role in chemical-induced autophagy [21,60]. In the current study, ZnO NPs exposure induced autophagy of mouse Leydig TM3 cells, which could be inhibited by NAC, a scavenger of ROS. Collectively, these results provide clear evidence that oxidative stress was critical in ZnO NPs-induced autophagy in mouse Leydig TM3 cells.

**Figure 7:**
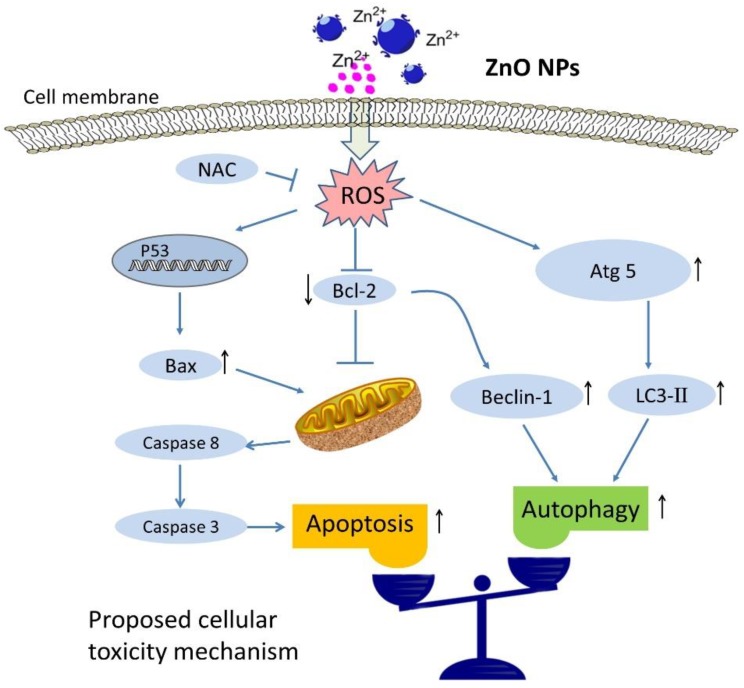
Schematic representation of the activation mechanism of apoptosis and cytoprotective autophagy in mouse Leydig cells after ZnO NP exposure. The up-regulation expression of protein is indicated by up arrow (↑), and down-regulation expression is indicated by down arrow (↓) in schematic illustration.

Both cell survival and death can be related to autophagy when the cells are subject to stressful conditions. In most circumstances, autophagy will promote cell survival [35,61]. However, autophagy is also considered to be a form of non-apoptotic programmed cell death—“type II” or “autophagic” cell death [62,63]. To investigate the role of autophagy in ZnO NPs-induced apoptosis, apoptosis was measured after the treatment of ZnO NPs in the absence or presence of autophagy inhibitor. Surprisingly, inhibition of autophagy could further induce apoptosis of mouse Leydig TM3 cells (Figure 7). These results illustrate that autophagy plays a cytoprotective role in ZnO NPs-induced apoptosis of mouse Leydig TM3 cells.

## 4. Materials and Methods

### 4.1. Reagents

ZnO NPs (No. 721077), N-acetyl-L-cysteine (A7250), 3-Methyladenine (M9281), and Wortmannin (12-338) were obtained from Sigma (St. Louis, MO, USA). Mouse Leydig cell line (TM3) was obtained from the Cell Culture Center of the Institute of Basic Medical Science, Chinese Academy of Medical Sciences (Beijing, China). Anti-Caspase-3 (sc-7148), anti-Caspase-8 (sc-7890), anti-Bax (sc-493), anti-Bcl-2 (sc-492), and anti-β-actin (sc-69879) were purchased from Santa Cruz Biotechnology (Santa Cruz, CA, USA). Anti-LC3 (PD014), anti-Atg5 (PM050), and anti-Beclin-1 (PD017) were gained from MBL Co. Ltd. (Nagoya, Japan). The AnnexinV-FITC/PI Apoptosis Kit (V13242) was purchased from Invitrogen Life Technologies (Waltham, MA, USA). Oxidation-antioxidation assay kits of malondialdehyde (MDA) (A003-1), glutathione (GSH) (A006-1), superoxide dismutase (SOD) (A001-1-1) and glutathione peroxidase (GSH-PX) (A005), testosterone Assay Kit (H090), and protease inhibitor cocktail (W060) were bought from Nanjing Jiancheng Bioengineering Institute (Nanjing, China).

### 4.2. Nanoparticles and Characterization

In characterization tests, ZnO NPs dispersed in sterile Milli-Q water (final concentration 1 mg/mL, Milford, MA, USA) were put into an ultrasound bath (100 W, Shanghai, China) to break up the aggregates before transmission electron microscopy (TEM, JEM-200CX, JEOL, Japan) and dynamic light scattering (DLS) analyses (Malvern Zeta sizer Nano ZS, Malvern Instruments, U.K.).

### 4.3. Animal Administration

Adult male Kunming mice (8 weeks old, 20–25 g) were obtained from the Shanghai Laboratory Animal Center, Chinese Academy of Sciences (CAS, Shanghai, China). The mice were housed in an isolated and air-conditioned animal room with water and rodent food supplement. All animals were acclimated to this environment for at least one week prior to the experiment. Experiments were approved by the Animal Ethics Committee of Nanchang University, China, SYKX2015-0001, 12 October 2015. The mice were intragastrically (i.g.) administered with ZnO NPs (0, 100, 200, 400 mg/kg/day, diluted in water) for 28 days and were then anesthetized with carbon dioxide inhalation, followed by cervical dislocation. The serum samples were collected following standard operation procedures. Then, the testes and the epididymis were quickly dissected free of fat, decapsulated, and frozen in liquid nitrogen.

### 4.4. Histology

Male mouse testis and epididymis tissues were stained with hematoxylin and eosin (HE) according to the method described by Chen et al. [43].

### 4.5. Western Blotting Analysis

The homogenized testis tissue and mouse Leydig TM3 cells were harvested in lysis buffer (50 mM Tris pH 7.5, 0.3 M NaCl, 5 mM EGTA, 1 mM EDTA, 0.5% Triton X-100, 0.5% NP40) containing protease inhibitor cocktail. Then, the supernatants were collected for Western blot after centrifuge for 10 min at 12 000× *g*. All primary antibodies and their recommended secondary antibodies were diluted 1:1000 and 1:5000, respectively.

### 4.6. Detection of Testosterone Content

The testosterone level in the serum was determined by ELISA kit according to the manufacturer’s instructions (Nanjing Jiancheng Bioengineering Institute, Nanjing, China).

### 4.7. Cell Culture and ZnO NP Treatment

Mouse Leydig TM3 cells were cultured at 37 °C in a 5% CO_2_ atmosphere in Dulbecco’s modified Eagle’s medium (DMEM, Gibco, Langley, OK, USA), supplemented with 5% horse serum (Gibco, Langley, OK, USA) and 2.5% fetal bovine serum (FBS, Gibco, Langley, OK, USA). The cultured cells were seeded and incubated for 24 h before exposure to varying concentrations of ZnO NPs or ZnCl_2_ as per experimental designs. The summary of the research design was illustrated in Figure S1.

### 4.8. Cell Viability Assay

The cells were seeded at a density of 1 × 104 cells per well in medium in 96-well plates and incubated for 24 h. The medium was then replaced with ZnO NPs of indicated concentrations in the presence or absence of 5 mM NAC, 10 mM 3-MA, or 1 μM Wortmannin for 24 h. Cell viability was determined by measuring the absorbance at 570 nm after the cells were incubated with 0.5 mg/mL MTT in medium for 4 h.

### 4.9. AnnexinV-FITC/PI Apoptosis Assay

Apoptosis was determined by using an AnnexinV-FITC/PI Apoptosis Kit from Invitrogen Life Technologies (Waltham, MA, USA) as described previously [64].

### 4.10. Oxidative Stress Measurement

The resultant supernatants of homogenized mouse Leydig TM3 cells were utilized to determine the activities of GSH-PX and SOD and the levels of GSH and MDA by using the commercial kits following the manufacturer’s instructions. The protein concentration was detected by the Bradford assay.

### 4.11. Transmission Electron Microscopy (TEM) Analysis

Mouse Leydig TM3 cells were treated with ddH_2_O, 4 μg/mL ZnO NPs or 5 mM NAC, plus 4 μg/mL ZnO NPs for 24 h. Then, the autophagic vacuoles were observed by TEM as previously described [65]. The cells treated for 2 h by starvation media (140 mM NaCl, 1 mM CaCl_2_, 1 mM MgCl_2_, 5 mM glucose, and 20 mM HEPES at pH = 7.4 supplemented with 1 % BSA) were used as the positive control of autophagy.

### 4.12. Statistical Analysis

The data were represented as means ± SE. Statistical analyses were performed using a one-way ANOVA with Newman–Keuls multiple range test. *p* < 0.05 was considered statistically significant.

## 5. Conclusions

ZnO NPs could cause disruption and atrophy of seminiferous epithelium, and even damage to spermatogenesis in male mice. Apoptosis and autophagy were induced by ZnO NPs in the testis tissue with a decreased level of serum testosterone. In vitro studies demonstrated that ZnO NPs markedly inhibited the viability of mouse Leydig TM3 cells and induced apoptosis and autophagy. Oxidative stress was also induced after the cells were treated with ZnO NPs, while inhibition of oxidative stress could rescue the induction of apoptosis and autophagy, indicating that oxidative stress was involved in ZnO NPs-induced apoptosis and autophagy. However, suppression of autophagy further inhibited cell viability with increase of the apoptosis levels. Taken together, we have provided detailed evidence that oxidative stress is involved in ZnO NPs-induced apoptosis and autophagy of mouse Leydig TM3 cells, while autophagy contributes to counteract the reproductive toxicity of ZnO NPs in the testis.

## Supplementary Materials

Supplementary materials can be found at https://www.mdpi.com/1422-0067/20/16/4042/s1.
