# Supplementary material for: Role of Autophagy in Zinc Oxide Nanoparticles-Induced Apoptosis of Mouse LEYDIG Cells
Source: Int J Mol Sci. 2019 Aug 19;20(16):4042. doi: 10.3390/ijms20164042 (PMC6720004; doi:10.3390/ijms20164042)
Supplement: Supplementary file 1 [file ijms-20-04042-s001.pdf]

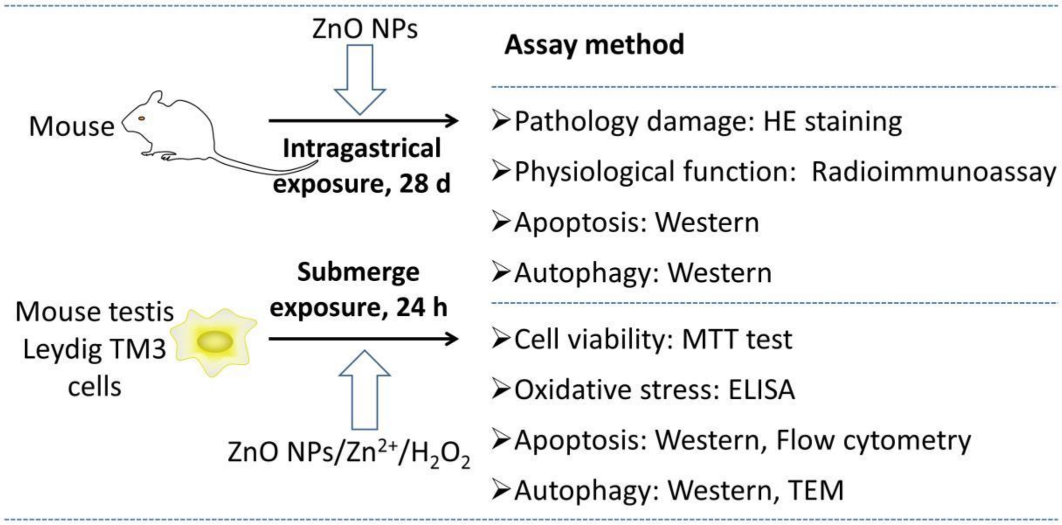


**Figure S1.** Schematic diagram of the experiment tal design.

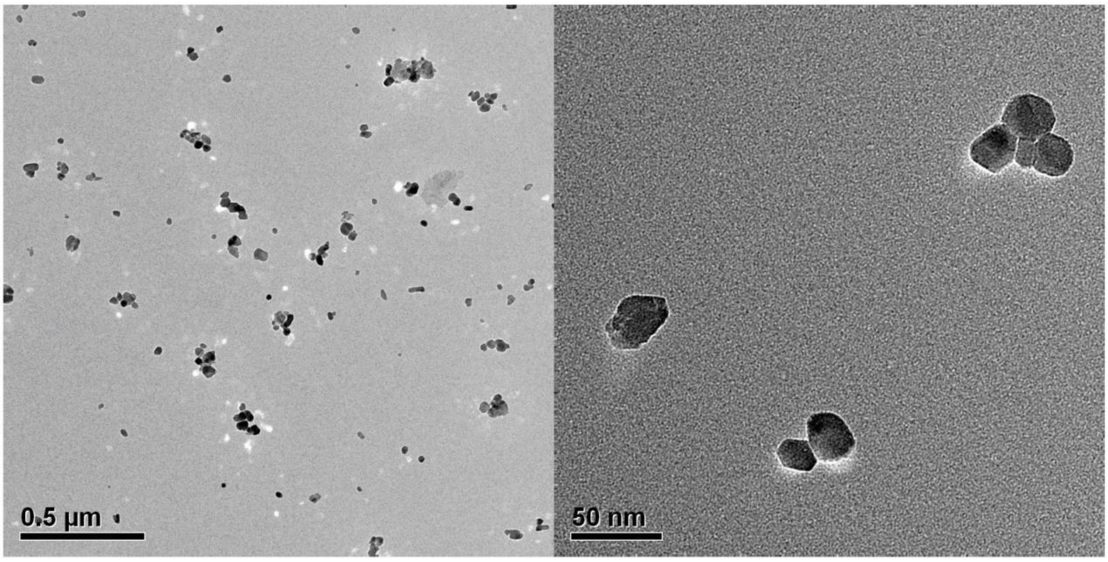


**Figure S2.** Transmission electron microscopy (TEM) images of ZnO NPs.

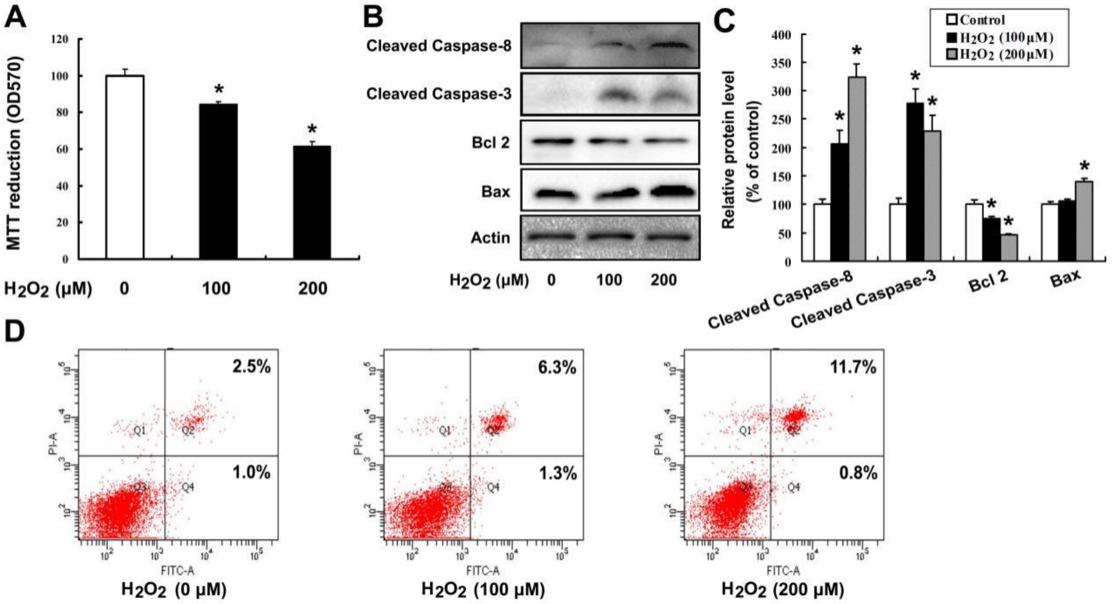


**Figure S3.** Oxidative stress induced apoptosis of mouse Leydig TM3 cells. Mouse Leydig TM3 cells were treated with 0–200 μM H2O2 for 24 h; then, cell viability (**A**), the protein levels of cleaved Caspase-8, cleaved Caspase-3, Bcl 2 and Bax (**B**) and the AnnexinV-positive staining cells (**D**) were tested by MTT assay, Western blot and flow cytometry, respectively. (**C**) The relative protein levels of cleaved Caspase-8, cleaved Caspase-3, Bcl 2 and Bax were quantified by densitometry. The experiment was done in triplicate and repeated for three times. Data were analyzed by one-way ANOVA. * $p < 0.05$.

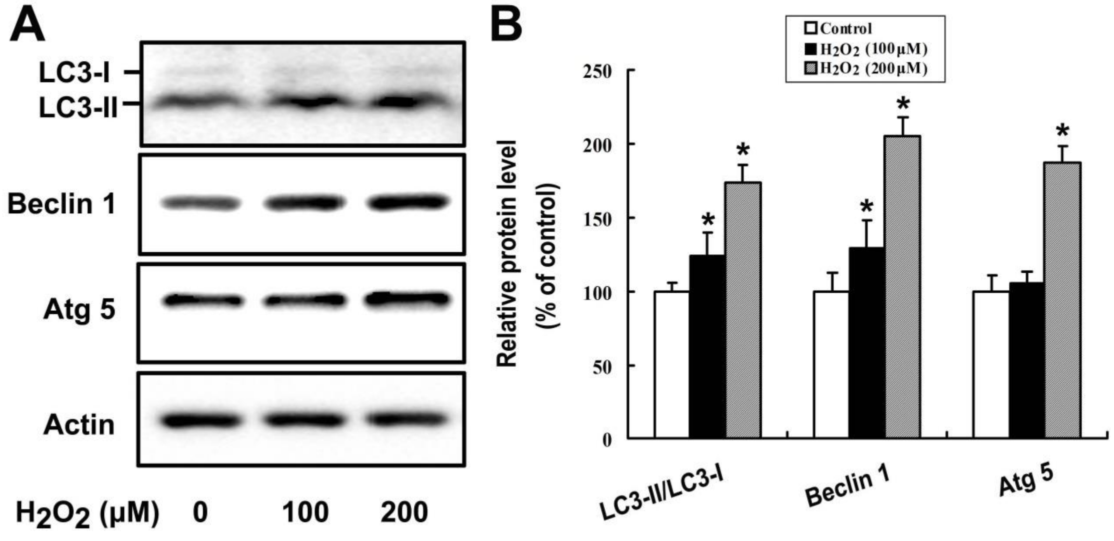


**Figure S4.** Oxidative stress induced autophagy of mouse Leydig TM3 cells. (**A**) Mouse Leydig TM3 cells were treated with 0–200 μM H2O2 for 24 h; then, the protein levels of LC3, Atg5 and Beclin1 were quantified by Western blot; Actin was used as an internal control. (**B**) The relative protein levels of LC3, Atg5 and Beclin1 were quantified by densitometry. The experiment was done in triplicate and repeated for three times. Data were analyzed by one-way ANOVA. * $p < 0.05$.

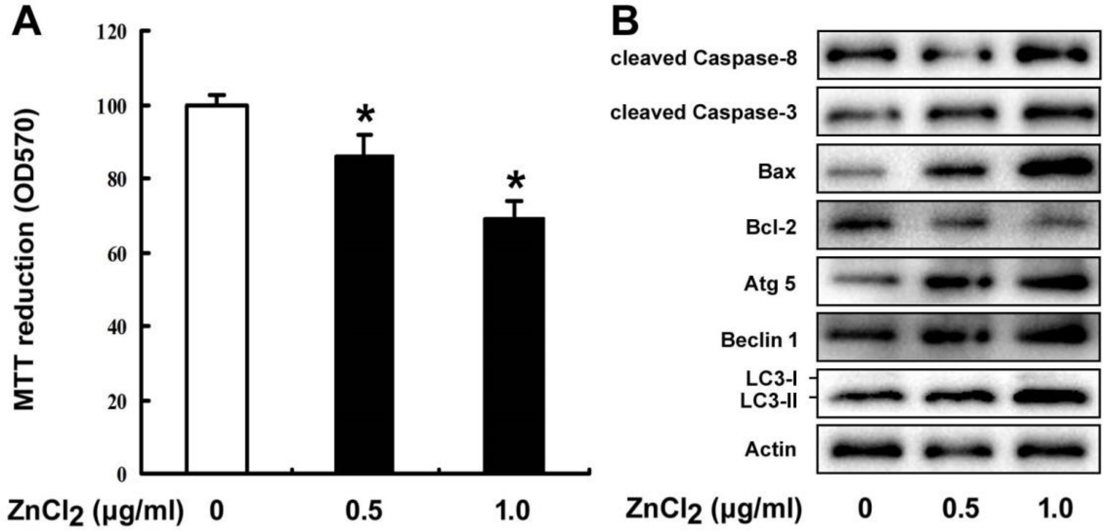


**Figure S5.** ZnCl2 induced apoptosis and autophagy of mouse Leydig TM3 cells. Mouse Leydig TM3 cells were treated with 0–1 μg/mL ZnCl2 for 24 h; then cell viability (**A**) and the levels of apoptosis and autophagy related proteins (**B**) were determined by MTT assay and Western blot, respectively. The experiment was done in triplicate and repeated three times. Data were analyzed by one-way ANOVA. * $p < 0.05$.
